# First-in-Man Use of Sutureless Perceval Valve for Endocarditis After Ozaki Procedure: A Bail-Out Strategy in Redo Infectious Aortic Valve Surgery

**DOI:** 10.3390/reports9010033

**Published:** 2026-01-24

**Authors:** Ziyad Gunga, Jorge Sierra, Guillaume Fahrni, Carlo Marcucci, Matthias Kirsch

**Affiliations:** 1 Department of Cardiovascular Surgery, Lausanne University Hospital, 1011 Lausanne, Switzerland; jorge.sierra@chuv.ch (J.S.); matthias.kirsch@chuv.ch (M.K.); 2 Department of Radiology, Lausanne University Hospital, 1011 Lausanne, Switzerland; guillaume.fahrni@chuv.ch; 3 Department of Anesthesiology, Lausanne University Hospital, 1011 Lausanne, Switzerland; carlo.marcucci@chuv.ch

**Keywords:** Ozaki, endocarditis, sutureless valve, Perceval, aortic valve replacement

## Abstract

**Background and Clinical Significance**: The Ozaki procedure offers excellent hemodynamics and mid-term durability, but infective endocarditis (IE), although rare, remains its most serious complication and frequently requires complex redo surgery. Sutureless valve technology, particularly the Perceval bioprosthesis, has shown value in high-risk endocarditis due to reduced annular manipulation and rapid deployment. **Case Presentation**: We describe the first reported case of Perceval sutureless valve implantation as a bail-out strategy for IE after a prior Ozaki procedure. A 68-year-old male previously treated with Ozaki reconstruction and LIMA-LAD bypass presented with septic and cardiogenic shock caused by Streptococcus bovis endocarditis, two years after the first surgery. TOE revealed torrential aortic regurgitation from destruction of the anterior neocuspid and large vegetations. Despite a EuroSCORE II of 89.5%, emergent redo surgery was undertaken. Redo sternotomy revealed extensive leaflet destruction and a sub-annular abscess involving two sinuses. Following radical debridement and annular reconstruction, a medium Perceval valve was implanted due to severe tissue fragility. The prosthesis seated securely with no paravalvular leakage. **Conclusions**: This case demonstrates that the Perceval sutureless valve can be an effective bailout option for post-Ozaki infective endocarditis, particularly when annular integrity is compromised, and conventional sutured prostheses are high risk. The combination of rapid deployment and minimal annular stress may expand therapeutic possibilities in complex redo aortic surgery.

## 1. Introduction and Clinical Significance

Since its development, the Ozaki procedure, an autologous pericardial aortic valve reconstruction, has steadily gained recognition as a landmark innovation in aortic valve surgery. Initially conceived for younger patients to optimize valve hemodynamics and eliminate the need for prosthetic material, its indications have broadened considerably. Today, it is increasingly adopted in older and high-risk populations, offering a compelling alternative to conventional valve replacement thanks to its excellent mid-term outcomes, native-like valve dynamics, and the absence of prosthetic-related complications. Among the potential drawbacks, infective endocarditis (IE) remains the most serious and life-threatening complication of this technique. Although rare, IE carries a significant clinical burden and frequently necessitates complex reintervention in hostile anatomical environments. In a recent large single-center series from Toho University Ohashi Medical Center (2025), Fujikawa-Osaki et al. identified 40 reoperations due to endocarditis among 1333 patients treated with the Ozaki procedure, yielding an incidence of 0.45% per patient-year [1]. This underscores both the rarity and clinical gravity of such a complication. Concurrently, the emergence of sutureless valve technology, particularly the Perceval Sutureless bioprosthesis (Corcym, Saluggia, Italia), has reshaped the approach to complex aortic valve reoperations [2]. As noted by Elgharaby et al. [3], the Perceval valve is being increasingly utilized in endocarditis settings, particularly in cases involving degenerated or infected prosthetic valves. Thanks to its inherent advantages, including shortened cross-clamp time, streamlined deployment, and minimal annular manipulation, it is particularly valuable in fragile, inflamed, or infected tissue beds, where conventional suturing techniques risk paravalvular leakage or annular injury.

We present the first-in-man case to our knowledge of Perceval valve implantation as a bail-out solution for infective endocarditis following prior Ozaki reconstruction in a critically ill patient. This unique scenario highlights the potential synergy between two innovative techniques and expands therapeutic options for managing complex infective aortic pathology in the re-operative setting.

## 2. Case Presentation

**Figure 1:**
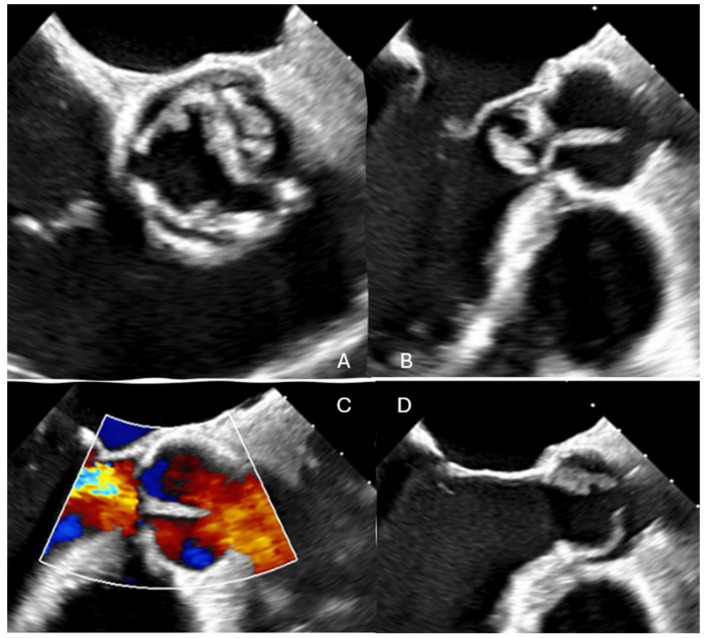
Pre-operative trans-esophageal echocardiography revealed severe aortic regurgitation (**C**) resulting from extensive destruction of the non-coronary cusp (**A**,**B**,**D**), accompanied by multiple mobile vegetations consistent with active infective endocarditis (**A**,**B**,**D**).

A 68-year-old male with a previous surgical history of Ozaki aortic valve reconstruction and LIMA-LAD coronary artery bypass grafting in May 2023 for bicuspid aortic valve disease presented in the emergency with acute decompensated heart failure. Blood cultures yielded Streptococcus bovis, and transesophageal echocardiography (TOE) revealed torrential aortic regurgitation (EROA > 80 mm^2^ and regurgitant volume > 80 mL) due to destruction of the anterior neocuspid leaflet, with associated large, mobile vegetations (Figure 1) (Supplementary Material S1)

**Figure 2:**
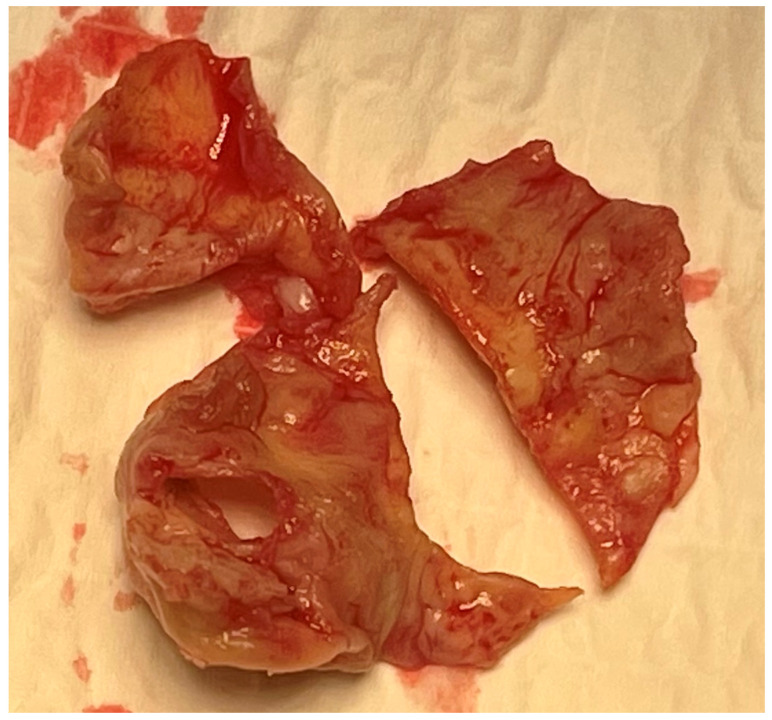
Severely destroyed autologous pericardial neocusps after prior Ozaki reconstruction, with marked leaflet thickening and inflammatory changes. One cusp shows a large central fenestration, leading to loss of coaptation and explaining the severe aortic regurgitation.

**Figure 3:**
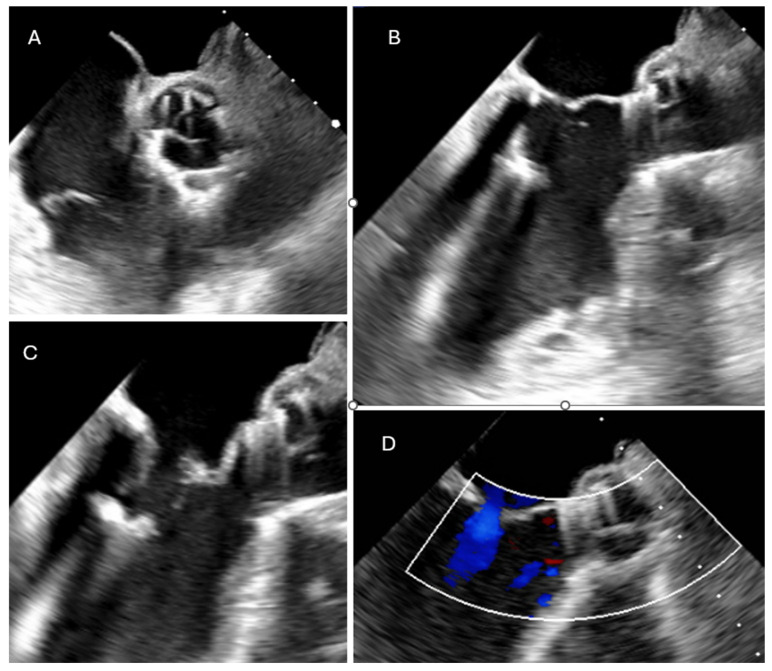
(**A**–**C**) Post-operative TOE following cusp and vegetation excision, annular debridement, and implantation of a Perceval Sutureless valve, showing a well-seated prosthesis without any paravalvular leak (**D**).

**Figure 4:**
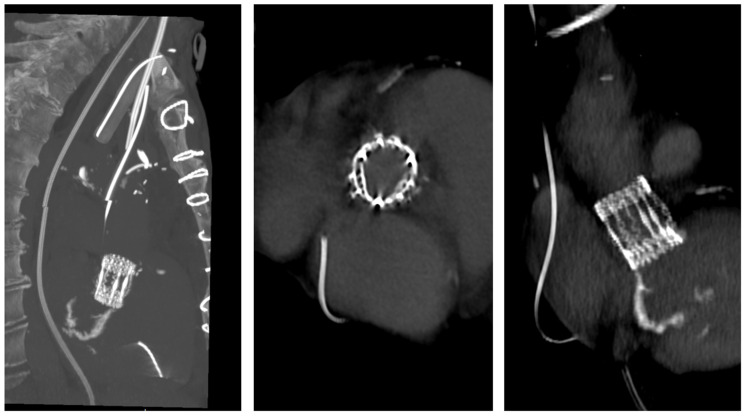
Post-operative Three-dimensional CT reconstruction demonstrating the Perceval sutureless valve in optimal position.

The patient experienced rapid clinical deterioration, progressing to multiorgan failure characterized by septic and cardiogenic shock (lactate level at 7 mmol/L), acute hepatic dysfunction (shock liver), acute renal failure, septic encephalopathy, disseminated intravascular coagulation, and new-onset atrial flutter. The preoperative LV function was assessed between 15 and 20%, under inotropic support by dobutamine at 500 microgram/min and vasoactive drugs like norepinephrine and vasopressin (Table A1). Despite a calculated in-hospital mortality risk of 89.5% based on the EuroSCORE II, the severity of the clinical scenario mandated immediate salvage surgery. A redo median sternotomy was undertaken in a technically complex setting, complicated by the absence of the pericardium and dense mediastinal adhesions from the previous surgery. Careful and meticulous dissection allowed for the safe identification of the great vessels. Central cannulation was achieved via the aortic arch for arterial return and bicaval venous access for drainage. Cardiopulmonary bypass (CPB) was initiated under normothermic conditions, ensuring adequate systemic perfusion. The aorta was cross-clamped, and a transverse aortotomy was performed. Myocardial protection was provided through the administration of cold blood cardioplegia directly into the coronary ostia, with repeated doses to maintain optimal myocardial preservation throughout the procedure. Intraoperative exploration revealed extensive destruction: the left neocuspid leaflet was completely perforated (Figure 2), and a large, highly mobile vegetation was found on the ventricular aspect of the valve. Furthermore, a subannular abscess was identified, extending into the subcommissural triangular space between the left and right aortic cusps. Radical debridement of all infected and necrotic tissue was undertaken, with evacuation of purulent material. The abscess cavity was meticulously excised until only macroscopically healthy tissue remained. The resulting defect, involving the annular region and extending approximately one centimeter into both sinuses, was reconstructed using two layers of continuous 4-0 Prolene sutures. Pericardial patch reinforcement was not considered appropriate due to the rigidity of the reconstruction and the potential increased risk of infection associated with the introduction of foreign material. Given the fragility of the annular tissue and the extensive debridement required, the use of a conventional sutured bioprosthesis was deemed inadvisable. A medium-sized (22–23 mm) Perceval sutureless bioprosthetic valve was therefore selected and deployed successfully. The aortotomy was closed using an everting mattress suture technique with two layers of 5-0 Prolene sutures. Weaning from CBP proved challenging due to biventricular dysfunction in the context of preoperative multiorgan failure. As a result, femoro-femoral veno-arterial extracorporeal membrane oxygenation (VA-ECMO) was instituted intraoperatively to provide circulatory support and facilitate end-organ recovery and persistent hemodynamic instability. The total cross-clamp time was 58 min, whilst the CBP time was 125 min. Hemodynamic stabilization was gradually achieved, and ECMO was successfully explanted after 5 days. The patient remained in the intensive care unit for three weeks, during which progressive clinical improvement was observed. Transthoracic echocardiography performed prior to ICU discharge demonstrated normal function of the implanted Perceval valve (Figure 3 and Figure 4), with no evidence of paravalvular leakage or residual vegetations, with an ejection fraction of 35%. At the three-month follow-up, the patient remained clinically stable, and repeat imaging confirmed preserved valve function (Peak and mean transvalvular gradients of 15 and 8 mmHg, respectively) without signs of recurrent endocarditis (Figure 3 and Figure 4).

## 3. Discussion

Among the rare but serious complications of the Ozaki procedure, infective endocarditis (IE) emerges as the leading cause of reoperation. While the Ozaki technique was designed to minimize foreign material and thus reduce the risk of infection, clinical experience has not consistently confirmed this protective effect. The most comprehensive data from Toho University Ohashi Medical Center, where the procedure was first developed, report an annual IE incidence of 0.45%, with IE responsible for 89% (40/45) of reoperations in a cohort of 1333 patients [1]. Similar findings were echoed by Unai et al. [4], Osaki et al. [5], and Iida et al. [6], who observed that IE not only persists as a risk after Ozaki but also overwhelmingly constitutes the primary indication for reintervention. Notably, the use of autologous pericardium did not confer a reduced risk of infection compared to standard prosthetic valves like PERIMOUNT used as in the control group in the study led by Unai et al. [4].

Outside Japan, multicenter and single-institution series show variable rates of IE after the Ozaki procedure, ranging from 0% [7,8] to approximately 1% per year [9]. Although some cohorts, such as those by Patel et al. [10] and Prinzing et al. [11], reported isolated IE cases requiring redo procedures, overall trends confirm that infective endocarditis, when it occurs, poses a significant therapeutic challenge, particularly in the context of tissue destruction, annular distortion, and fragile autologous cusps.

In this landscape, reoperation strategies remain a subject of debate, especially in patients with structural valve failure complicated by active endocarditis. Our case highlights an innovative and pragmatic solution: the use of a Perceval sutureless valve as a bailout strategy after failure of the Ozaki procedure due to IE in a high-risk surgical patient. To date, only one case has been reported of Perceval implantation following an Ozaki procedure [12], but that case did not involve an infectious context. Our report is, to our knowledge, the first to describe successful Perceval implantation in a patient with post-Ozaki infective endocarditis.

The use of Perceval in endocarditis is increasingly documented. Several authors [13,14,15,16,17,18] have reported favorable outcomes with sutureless valves in high-risk endocarditis settings, including valve-in-valve and redo operations. The main arguments in favor of Perceval in these contexts include its rapid deployment, shorter cardiopulmonary bypass and cross-clamp times, and reduced need for annular suturing, particularly advantageous when native tissue is inflamed, friable, or debrided. Rosello-Diez et al. [14] and Zubarevitch et al. [15] emphasized its utility in cases with extensive destruction, where conventional sutured prostheses may be technically hazardous. Although perioperative mortality in these series remained significant (up to 23%), this reflects the severity of the underlying disease and the high-risk nature of the patient population rather than the limitations of the device itself.

In our case, after careful excision of infected tissue and assessment of annular integrity, Perceval implantation offered a technically safe and time-efficient solution with excellent immediate hemodynamics and no signs of recurrent infection or paravalvular leakage at follow-up. This aligns with the broader findings from Ozgur et al. [16], who supported Perceval’s role as a viable alternative to conventional bioprostheses in endocarditis scenarios.

Importantly, the mid-term infective risk profile of the Perceval valve compares favorably with both conventional bioprostheses and the Ozaki procedure. In a five-year follow-up study, Shrestha et al. [19] reported an endocarditis incidence of 1.9% following Perceval implantation, corresponding to approximately 0.4% per patient-year, like the rates observed in transcatheter aortic valve implantation (TAVI), where Ruchonnet et al. [20] described an annual incidence of 0.5%. These figures are also comparable to the infective risk reported in the large Ozaki series. Together, these data support the notion that sutureless valves like Perceval may serve not only as effective bail-out options in technically challenging or infected fields but also as reliable and durable solutions with an acceptable long-term infective profile.

While the Perceval sutureless valve offers distinct advantages in selected high-risk scenarios, its use is not universally applicable and carries important limitations. The prosthesis requires a relatively circular and symmetric annulus for optimal anchoring and function [21,22]; significant annular distortion or asymmetric calcification may compromise sealing and increase the risk of paravalvular leak or device malposition [23,24]. In addition, the Perceval valve is not suitable for annular diameters outside the manufacturer’s approved range (typically 19–27 mm) [25], limiting its applicability in patients with extreme annular sizes. In cases of severe aortic root dilatation or fragile aortic wall integrity, particularly when reconstruction of the sinuses or root replacement is necessary, the device may not provide sufficient structural support or flexibility [25,26,27,28,29]. Finally, concerns have been raised regarding potential risks of conduction disturbances and pacemaker implantation [30,31], particularly in smaller annuli or heavily calcified valves. These factors should be carefully weighed during preoperative planning, and alternative strategies such as conventional sutured bioprostheses or homograft implantation, as reported, for example, by Komarov et al. in 2021 [32], may be preferable in anatomically unsuitable or structurally unstable cases, at the cost of a prolonged clamping and CPB time.

In post-Ozaki infective endocarditis, redo aortic valve surgery is typically performed in a hostile anatomical environment marked by leaflet destruction, annular inflammation, and frequent sub-annular abscess formation, all of which significantly complicate conventional valve replacement strategies. Although homograft root replacement has long been considered a reference option in destructive infective endocarditis, it is surgically demanding, dependent on graft availability, and associated with prolonged cross-clamp and cardiopulmonary bypass times: factors that may be poorly tolerated in unstable, high-risk patients [32]. In contrast, the Perceval sutureless bioprosthesis offers several decisive advantages in this setting. Its anchoring mechanism relies primarily on radial expansion rather than circumferential annular suturing, thereby minimizing additional trauma to friable or partially reconstructed tissue and reducing the risk of annular tearing or paravalvular leakage following radical debridement [14,15]. This feature is particularly relevant after abscess evacuation extending into the sinuses of Valsalva, as observed in our case. Furthermore, multiple series have demonstrated that sutureless valve implantation in active infective endocarditis is feasible and reproducible, with the added benefit of significantly shortened cross-clamp and cardiopulmonary bypass durations; an important determinant of outcome in patients presenting with septic or cardiogenic shock [13,14,15,16,17,18]. Importantly, mid-term data indicate that Perceval valves provide hemodynamic performance and durability comparable to conventional bioprostheses, with reinfection rates in the range of 0.4–0.5% per patient-year, like those reported after the Ozaki procedure and transcatheter aortic valve implantation [1,19,20]. Collectively, these data support the role of the Perceval sutureless valve as a reliable and pragmatic bailout option in post-Ozaki infective endocarditis, particularly when annular integrity is compromised, and conventional sutured prostheses or homografts would entail excessive technical risk or operative time. Although this report describes a single case, it highlights a potential surgical option that warrants further evaluation in larger series.

## 4. Conclusions

Given the growing global uptake of the Ozaki procedure and the predictable, although rare, occurrence of endocarditis-related failure, the availability of a safe and efficient bailout strategy is essential. Our case supports the notion that the Perceval valve may fulfill this role, especially in patients with annular damage, calcification, or tissue friability that complicates conventional valve reimplantation.

## Data Availability

The original data presented in the study are included in the article, further inquiries can be directed to the corresponding author.

## Supplementary Materials

The following supporting information can be downloaded at: https://www.mdpi.com/article/10.3390/reports9010033/s1, Supplementatry Material S1: Trans-oesophageal echography showing a torrential regurgitation.

## Appendix A

**Table A1:** Chronological overview of the patient’s clinical course.

Date	Event
**12 May 2023**	Single arterial CABG to the mid-LAD and aortic valve reconstruction (Ozaki procedure)
**8 May 2025**	Acute respiratory failure with syncope and low-flow state; brief CPR; Emergency and ICU admission
**8 May 2025**	Cardiogenic shock; TEE demonstrating severe aortic regurgitation due to infective endocarditis with suspected abscess
**9 May 2025**	Redo aortic valve replacement and abscess debridement and Perceval implantation with peripheral VA-ECMO support
**14 May 2025**	Successful ECMO weaning
**15 May 2025**	Discontinuation of inotropic support, continuation of targeted endocarditis treatment
**30 May 2025**	Discharge from ICU/transfer to readaptation
**27 August 2025**	TOE: preserved valve function, no sign of recurrent endocarditis
**10 December 2025**	Clinically well with TTE showing normal valve function
